# Development and Validation of the Death Pronouncement Burden Scale for Oncology Practice

**DOI:** 10.1089/pmr.2021.0082

**Published:** 2022-04-08

**Authors:** Yusuke Hiratsuka, Mitsunori Miyashita, Yu Uneno, Kiyohumi Oya, Soichiro Okamoto, Takaomi Kessoku, Hironori Mawatari, Shunsuke Oyamada, Junko Nozato, Keita Tagami, Akira Inoue

**Affiliations:** ^1^ Department of Palliative Medicine, Tohoku University School of Medicine, Sendai, Japan.; ^2^ Department of Palliative Medicine, Takeda General Hospital, Aizu Wakamatsu, Japan.; ^3^ Department of Palliative Nursing, Tohoku University School of Medicine, Sendai, Japan.; ^4^ Department of Therapeutic Oncology, Graduate School of Medicine, Kyoto University, Kyoto, Japan.; ^5^ Department of Internal Medicine, Palliative Care, Peace Home Care Clinic, Shiga, Japan.; ^6^ Department of Internal Medicine, Palliative Care, Medical Corporation Teieikai Chiba Home Care Clinic, Chiba, Japan.; ^7^ Department of Palliative Medicine, Yokohama City University Hospital, Yokohama, Japan.; ^8^ Department of Palliative and Supportive Care, Yokohama Minami Kyosai Hospital, Yokohama, Japan.; ^9^ Department of Biostatistics, JORTC Data Center, Tokyo, Japan.; ^10^ Department of Internal Medicine, Palliative Care, Tokyo Medical and Dental University Hospital, Tokyo, Japan.

**Keywords:** assessment instrument, clinician's burden, death pronouncement, oncology practice

## Abstract

**Background:**

Informing families of a patient's death is one of the most challenging responsibilities of clinicians who provide care for terminally ill patients. Although death pronouncement can be a highly stressful event for clinicians, no previous study has reported qualitative characteristics of the burden experienced by clinicians related to death pronouncements. Moreover, no scale has been developed to assess this burden.

**Objective:**

This study sought to develop a scale to evaluate clinicians' burden related to death pronouncement (Death Pronouncement Burden Scale for oncology practice [DPBS-oncol]) and examine its reliability and validity in Japan.

**Methods:**

We presented the DPBS-oncol to clinicians involved in oncology practice and examined its reliability and discriminant validity. To investigate the test-retest reliability of the scale, the DPBS-oncol was presented a second time to a subsample of the clinicians.

**Results:**

Factor analysis required a grouping of the 15 DPBS-oncol items into one factor. Cronbach's α coefficient of the total score of DPBS-oncol was 0.94, and the intraclass correlation coefficient of the total score of DPBS-oncol was 0.89. Regarding discriminant validity, DPBS-oncol total score was moderately correlated with other available scales for assessing clinicians' attitudes to end-of-life care.

**Conclusion:**

This study was the first to develop a scale to evaluate clinicians' burden related to death pronouncement. The DPBS-oncol, which includes 15 items, was validated and shown to have sufficient reliability.

## Introduction

Informing families of a patient's death is one of the most challenging responsibilities of clinicians who provide care to patients with terminal illness. Death pronouncement can be a highly stressful event for clinicians, particularly those who are younger or less experienced.^1–3^ The method of communication during the death pronouncement can affect a family's acute and long-term psychological well-being.^4^ Death pronouncement should be a compassionate communication with families, and an appropriate pronouncement can help to alleviate families' grief.^2,5^ Moreover, appropriate death pronouncement practice itself can be considered a type of bereavement care for family members.^6,7^ However, one previous study suggested that death pronouncement procedures were judged as acceptable in only 35% of cases.^8^

To deal with this challenging task, several education programs for death pronouncement have been developed.^9–11^ Several studies have reported the efficacy of educational interventions for death pronouncement.^5,12–16^ However, no scales for assessing the efficacy of these educational programs have been developed, and each previous study used a different assessment method. In addition, there are currently no scales for assessing clinicians' attitudes regarding death pronouncement, despite the development of several scales to assess clinicians' attitudes toward end-of-life care, including the Frommelt Attitude Toward Care of the Dying scale, Form B (FATCOD-Form B),^17,18^ the Scale of Confidence in Palliative Care,^19^ and the Palliative Care Self-Reported Practices Scale.^20^ However, none of these concurrent scales is focused on death pronouncement.

To date, death pronouncement has been reported to be the factor associated with clinicians' burden in end-of-life care because of both knowledge and technical issues.^1,21^ However, no previous study has reported qualitative characteristics of the burden experienced by clinicians in relation to death pronouncement, and no scale has been developed to assess this burden directly. We hypothesized that it was necessary to evaluate and improve the clinicians' burden related to death pronouncement, and the scale to assess the burden would be useful for evaluation of the efficacy of educational programs for death pronouncement. Therefore, we aimed to develop a scale for evaluating clinicians' burden related to death pronouncement (the Death Pronouncement Burden Scale for oncology practice [DPBS-oncol]) and to examine its reliability and validity in Japan.

## Methods

Development and validation phases were conducted for the questionnaire to make DPBS-oncol. In the current study, we focused on clinicians treating patients with cancer, who generally have a relatively predictable illness trajectory.^22^

### Development phase

The questionnaire was constructed to develop DPBS-oncol. First, we administered a semistructured interview with clinicians until no new relevant knowledge was being obtained from new participants (theoretical saturation). We interviewed 20 clinicians with more than three years of clinical experience who were involved in oncology practice. Participants were asked by a researcher (Y.H.) to describe how they felt regarding the burden related to death pronouncement. The semistructured interviews were based on an interview guide containing three questions (burden, difficulty, and mistake), which in turn was based in agreement by the researchers because no conceptual framework was available from past work, especially for Japan. The interviews were digitally audio-recorded, transcribed verbatim, and analyzed. Three authors (Y.H., Y.U., and S.O.) read and analyzed each interview transcript under the supervision of one author (M.M.). The interviews were analyzed using inductive thematic analysis in six stages: familiarization with data, generating initial codes, searching for themes, reviewing themes, defining and naming themes, and producing the report. Five main themes and 27 subthemes were identified by thematic analysis. Based on the findings, a draft questionnaire with 30 questions was created to evaluate clinicians' burden related to death pronouncement. The content validity (wording and format of the draft questionnaire) was assessed by discussions among researchers (Y.H., Y.U., S.O., and M.M.). Second, a web-based cognitive interview was conducted by one researcher (Y.H.) with same 20 participants as the semistructured interview, potential causes of missing data, and questions that might be difficult to answer. Third, a pilot study was conducted with 50 clinicians to evaluate the appropriateness of each item, and 15 items were selected for the validation phase.

### Validation phase

A web-based cross-sectional questionnaire using a Google form was completed anonymously by clinicians throughout Japan who care for patients with advanced cancer. We obtained the e-mail addresses of the participants at the same time to avoid and detect duplicate responses. We assured confidentially while collecting the e-mail addresses. The participating clinicians met the following eligibility criteria: to assess test-retest reliability, participants who spontaneously agreed to participate in the study completed a second web-based survey two weeks after the first. We set up the questionnaire so that participants could not submit the form until they had answered all of the questions.

### Participants and procedures

We identified potential participants among subscribers to mailing lists on palliative care, lung cancer, and general practice. The palliative care physician, oncologists, and general physicians were subscribed to the mailing lists. In Japan, clinicians with less than two years of experience rarely deliver death pronouncements. Thus, we defined the inclusion criteria as follows: (1) clinicians with more than three years of clinical experience who were involved in oncology practice and (2) clinicians who had experienced the task of death pronouncement. The researcher (Y.H.) sent e-mails to the mailing lists on palliative care, lung cancer, and general practice with the following information: (1) the purpose and methods of the survey and (2) a link to the web-based questionnaire. The subscriber who understood the purpose and methods of the survey answered the web-based questionnaire. Thus, we used a convenience sample. We considered the provision of answers to the questionnaire as consent to participate in the study.

### Measurements

We used the following scales to assess clinicians' attitudes toward end-of-life care for investigation of concurrent validity because there are no scales focused on death pronouncement.

The Frommelt Attitude Toward Care of the Dying scale, Form B, Japanese version (FATCOD-Form B-J), was based on Frommelt's original FATCOD.^17,18^ FATCOD was developed to examine the effect of an educational program on attitudes toward caring for terminally ill people and their families. FATCOD has been translated into Japanese and validated.^23^ FATCOD-Form B-J has 30 attributes with Likert-type questions using five scales (1 = strongly disagree, 2 = disagree, 3 = uncertain, 4 = agree, 5 = strongly agree) and consists of two domains: I, positive attitude toward caring for dying patients; and II, perception of patient- and family-centered care. We used domain Ⅰ (16 attributes) for this study.

Scale of Confidence in Palliative Care was developed in Japan to explore confidence in the ability to provide palliative care.^19^ The scale contains five items concerning confidence in providing palliative care on a 5-point Likert scale (1 = no difficulties, 2 = occasionally, 3 = sometimes, 4 = often, 5 = always).

The Palliative Care Self-Reported Practices scale was developed in Japan to assess the effectiveness of educational programs for general nurses.^20^ The scale comprises seven subscales (pain, dyspnea, delirium, dying-phase care, communication, and patient- and family-centered care). The scale assesses adherence to recommended practices in palliative care fields using a Likert-type scale (from 1, “never” to 5, “very much”; high values suggest a high level of performance of recommended practices). We used the domain “dying-phase care” in this study.

In addition to these scales, we asked participants about the burden associated with death pronouncement using a Likert-type scale (from 0, “never” to 10, “very much”; high values suggest a high level of burden for death pronouncement).

### Sample size

We estimated the necessary sample size on the basis of the Consensus Based Standards for the Selection of Health Measurement Instruments (COSMIN).^24^ The COSMIN requires an item number × 7 and more than 100 participants to be classified as “excellent.” Therefore, 105 participants (15 items × 7) were required in the current study, and the target sample was set at 120 participants with the expectation of some dropouts.

### Analysis

Analysis consisted of the following: (1) examination of the ceiling and floor effects for items on each scale and excluded items (cutoff: >90% of responses were 1 or 6 on the 6-point Likert-type scale) for item selection. (2) Bartlett's test of sphericity to examine the feasibility of factor analysis; (3) exploratory factor analysis (maximum likelihood method) to investigate item selection and factor validity after eliminating responses with a clear bias; (4) calculation of Cronbach's coefficient to investigate internal consistency; (5) a repeat survey and calculation of intraclass correlation coefficients (ICCs) for each factor score to investigate reliability; (6) the assumption of combinations between factors predicted to be correlated with the FATCOD-Form B-J, Scale Confidence Palliative Care, and Self-Reported Palliative Care Practices scale to investigate the concurrent validity of the DPBS-oncol. Pearson's correlation coefficients were calculated; and (7) investigation of known-group validity to determine whether the DPBS-oncol score was related to participants' characteristics using the Wilcoxon rank sum test. Statistical significance was set at the *p* < 0.05 level, and JMP version 16 for Windows (SAS, Cary, NC) was used for statistical analyses.

### Ethics

In accordance with the ethical guidelines for human research of Japan's Ministry of Health, Labor, and Welfare, informed consent was waived because of the nature of the study. We explained using a mailing list that participation in the survey was voluntary for each individual, that the survey forms were anonymous and that privacy would be maintained, and that answering the web-based questionnaire would be taken to indicate consent. The study obtained approval from the independent ethics committee of Tohoku University School of Medicine (approval no. 2020-1-768).

## Results

### Study sample

A total of 128 web-based questionnaires were returned at the first survey. No responses were excluded from analysis. Regarding the second survey as the retest, of the 128 questionnaires in the first survey, 111 (86.7%) questionnaires were returned and none was excluded from analysis. The characteristics of the respondents are summarized in Table 1.

**Table 1.** Participants' Characteristics and Scores of Each Scale (*n* = 128)

Characteristics	*n* (%)
Clinical experience (years, median [range])	11.5 (3–35)
Specialty	
Palliative care	52 (40.6)
Internal medicine	19 (14.8)
General physician	12 (9.4)
Medical oncology	10 (7.8)
Surgery	7 (5.5)
Respiratory medicine	6 (4.7)
Gynecology	5 (3.9)
Otolaryngology	5 (3.9)
Others	12 (9.4)
Death pronouncements per year (median [range])	17.5 (0–250)
FATCOD-Form B-J (mean ± SD)	65.0 ± 7.0
Scale Confidence in Palliative Care (mean ± SD)	15.5 ± 4.6
Self-Reported Palliative Care Practices (mean ± SD)	11.5 ± 2.4
Burden associated with death pronouncement (mean ± SD)	3.9 ± 2.3

FATCOD Form B-J, Frommelt Attitude Toward Care of the Dying scale, Form B, Japanese version; SD, standard deviation.

Table 1 also shows mean scores and standard deviations for each scale: FATCOD-Form B-J (domain Ⅰ), Scale of Confidence in Palliative Care, Palliative Care Self-Reported Practices scale (dying-phase care), and burden associated with death pronouncement.

### Item selection and factor validity

The results of the factor analysis are shown in Table 2. No items were excluded because of skewed response in 90% or more respondents. The *p*-values driven by Bartlett's test of sphericity were >0.05. Thus, exploratory factor analysis was conducted with the 15 items. The maximum likelihood method was used for factor extraction because of its attractive statistical properties. Items with factor loading <0.4 and communality <0.3 would be eliminated. After confirming the consistency of item contents, a single factor comprising 15 items was ultimately obtained. We did not perform rotation method because factor analysis extracted just one factor. The cumulative proportion of explanation of variance was 64.6%. Thus, the DPBS-oncol has 15 items with Likert-type questions using six scales (1 = strongly disagree, 2 = disagree, 3 = somewhat disagree, 4 = somewhat agree, 5 = agree, 6 = strongly agree), and the total score (range 15–90) was calculated by the sum of each item's score. In the DPBS-oncol responses, “strongly agree” indicated a high burden and “strongly disagree” indicated a low burden.

**Table 2.** Factor Analysis and Reliability of Death Pronouncement Burden Scale (*n* = 128)

Mean (SD), 50.5 (15.2); Cronbach's α coefficient = 0.94; ICC, 0.89	Factor loading	Communalities
I find it hard to explain the patient's death to his or her family	0.86	0.73
I feel awkward talking with the patient's family just after the death pronouncement	0.85	0.73
I feel nervous when I am asked to pronounce a patient's death	0.84	0.70
I feel uncertain about the death pronouncement procedure	0.83	0.70
I feel uncertain about when I should enter the patient's room for the death pronouncement	0.83	0.69
I find it difficult to decide when to pronounce the patient's death to his or her family	0.82	0.68
I rarely have the right words to comfort the family	0.81	0.66
I feel that it is hard to know what to say to the family in consideration of their emotions	0.80	0.63
I feel that I cannot pronounce a patient's death with confidence	0.77	0.60
I feel anxious about the patient's family members' personalities and how they will respond	0.77	0.59
I feel uneasy when I am asked to pronounce a patient's death	0.75	0.57
I feel unsure about whether I should talk with the patient's family after the death pronouncement	0.73	0.54
I feel unsure about whether I should talk with the patient's family before the death pronouncement	0.73	0.54
I am not confident enough to fill out the death certificate form correctly	0.72	0.52
I find it more difficult to pronounce a patient's death when I am not the patient's primary physician	0.68	0.46

Cumulative proportion, 64.6%.

ICC, intraclass correlation coefficient.

### Internal consistency and reliability

The internal consistency (Cronbach's α coefficient) and test-retest reliability (ICC) are shown in Table 2. A total of 111 patients responded to the second survey that was sent to assess reliability. Cronbach's α coefficient of the total score was 0.94, and the ICC of the total score was 0.89.

### Concurrent and discriminant validity

The concurrent and discriminant validity according to Pearson's correlation coefficients is shown in Table 3. DPBS-oncol total score was moderately correlated with the FATCOD-Form B-J (domain Ⅰ), Scale of Confidence in Palliative Care, Palliative Care Self-Reported Practices scale (dying-phase care), and the single item about clinicians' perceived burden associated with death pronouncement.

**Table 3.** Pearson's Correlation Coefficients between the Death Pronouncement Burden Scale Score and Other Scale Scores (*n* = 128)

	Total score
FATCOD-Form B-J	−0.64
Scale confidence in palliative care	−0.57
Self-reported palliative care practices	−0.50
Burden associated with death pronouncement	0.62

### Correlation with participants' characteristics

The known-group validity according to the Wilcoxon rank sum test results is shown in Table 4. Clinicians with a shorter duration of clinical experience (≤10 years), no palliative care specialty, and fewer experiences of death pronouncement (≤36 per year) had significantly higher DPBS-oncol scores.

**Table 4.** Known-Group Validity (*n* = 128)

	*N*	Total score	
		Mean ± SD	*p* ^*^
Clinical experience (years)			<0.01
≤10	58	48.6 ± 16.0	
>10	70	39.3 ± 15.1	
Specialty			0.01
Palliative care	52	39.2 ± 15.6	
Other	76	46.5 ± 15.9	
Death pronouncements per year			<0.01
≤36	95	45.8 ± 15.6	
>36	33	36.9 ± 16.1	

^*^ *p*-Values were calculated with the Wilcoxon rank sum test.

## Discussion

This study was the first to develop a scale for evaluating clinicians' burden related to death pronouncement. We developed the DPBS-oncol, which included 15 items covering important aspects of death pronouncement in Japan. The results confirmed that the DPBS-oncol exhibited sufficient validity and reliability. In addition, clinicians with shorter durations of clinical experience (≤10 years), no palliative care specialty, and fewer experiences of death pronouncement (≤36 per year) exhibited significantly higher DPBS-oncol scores.

On the basis of the factor analysis results, we identified a single factor that was associated with clinicians' burden related to death pronouncement. We assumed that clinicians' diathesis and families' diathesis could explain our results. Regarding clinicians' diathesis, the limited opportunities to learn methods for death pronouncement in practice may be reflected in the current results. Although several studies^5,12–16^ have examined educational programs for death pronouncement and reported their efficacy, no official educational program for death pronouncement has been conducted in Japan to date.^25,26^ The current finding that experienced clinicians exhibited lower DPBS-oncol scores suggests that an educational program for inexperienced clinicians might improve their confidence. Regarding families' diathesis, clinicians' psychological burden associated with communication with families during death pronouncement may have influenced our results. The methods of communication during death pronouncement can affect families' acute emotional responses and their long-term psychological well-being.^4^ Although death pronouncements can be a highly sensitive and stressful event for clinicians, the procedure can also be a valuable opportunity for compassionate communication with families and can help alleviate families' grief and emotional burden.^2,5^ Several studies^7,27,28^ conducted in Japan reported optimal methods of delivering the death pronouncement, and some of our participants may have been aware of this evidence. However, clinicians need to apply this evidence to individual families. Thus, we assumed that the distress involved in communication with families was associated with burden related to death pronouncement.

The reliability of the DPBS-oncol was assessed as “almost perfect” by ICC values and “excellent” by Cronbach's α values. Using the repeat survey method, the sample size in the first and second surveys was assumed to be sufficiently large. There might be less burden on participants when answering a web-based questionnaire compared with a paper-based questionnaire. Therefore, we considered that the reproducibility of the scale was sufficient.

The results of the concurrent and discriminant validity analyses were expected. We assumed that these concurrent scales such as FATCOD-Form B-J (domain Ⅰ), Scale of Confidence in Palliative Care, Palliative Care Self-Reported Practices scale (dying-phase care) and the burden associated with death pronouncement captured clinicians' attitudes toward end-of-life care, which includes death pronouncement. Therefore, the DPBS-oncol may be useful for accurately evaluating clinicians' attitudes toward end-of-life care.

Known-group validity was also examined. We found that inexperienced clinicians exhibited significantly higher DPBS-oncol scores. It is well known that death pronouncement is one of the most challenging clinical practices, particularly for younger clinicians.^1–3^ Clinicians other than palliative clinicians might have less experience of death pronouncement in daily practice. We assumed that palliative clinicians were experienced regarding death pronouncement and might experience less burden.

This study was the first to develop a scale for evaluating clinicians' burden related to death pronouncement. However, the current study involved several limitations. First, participants were recruited using convenience sampling of subscribers to mailing lists focused on palliative care, lung cancer, and general practice. Because convenience sampling was used, it is unclear whether our results are representative of the general population of clinicians. Thus, the heterogeneity might have affected the results. However, this was a validation study focused on correlations among scales and items, not on the representativeness of the sample or the distribution of the scale. Therefore, we do not believe that this is a critical flaw of the study. Second, only four relatively simple scales were used to assess concurrent and discriminant validity. Importantly, no previous scales have focused on death pronouncement, and the DPBS-oncol was moderately correlated with scores on scales that assess clinicians' attitudes to end-of-life care. Therefore, we believe that the DPBS-oncol exhibited sufficient construct validity. Third, although a sufficient sample size was used, development of the DPBS-oncol was exploratory. Therefore, confirmatory analysis with a larger population will be required to assess its long-term stability. Fourth, the results may have been affected by recall bias, if participants answered the DPBS-oncol on the basis of their most recent clinical experiences. Thus, responses may have been strongly influenced by participants' most recent death pronouncement experience. Therefore, we asked some participants to complete a second web-based survey two weeks after the first to minimize the recall bias in a retest. Fifth, there was a possibility that DPBS-oncol had contextual factors influencing measurement error. However, the contextual factors were unknown. Therefore, the study is needed to explore the contextual factors which might affect the measurement error mechanisms. Finally, the DPBS-oncol may specifically reflect Japanese clinicians' burden in relation to death pronouncement, and cultural differences may exist. Thus, it is currently unclear whether the DPBS-oncol is suitable for use with clinicians in other countries.

## Conclusions

The newly developed DPBS-oncol, which comprises 15 attributes, appears to provide an accurate measure of clinicians' burden related to death pronouncement. The DPBS-oncol has sufficient reliability and validity. Further studies will be needed to examine the efficacy of DPBS-oncol in capturing a meaningful change in education programs.

## Data Availability

The data that support the findings of this study are available from the corresponding author, Yusuke Hiratsuka, upon reasonable request. All authors agree to provide data to the journal for review if needed.

## Abbreviations Used

COSMIN: Consensus Based Standards for the Selection of Health Measurement Instruments
DPBS-oncol: Death Pronouncement Burden Scale for oncology practice
FATCOD-Form B: Frommelt Attitude Toward Care of the Dying scale, Form B
FATCOD-Form B-J: Frommelt Attitude Toward Care of the Dying scale, Form B, Japanese version
ICCs: intraclass correlation coefficients
SD: standard deviation
